# The Role of Magnetic Nanoparticles in the Localization and Treatment of Breast Cancer

**DOI:** 10.1155/2013/281230

**Published:** 2013-07-09

**Authors:** M. Ahmed, M. Douek

**Affiliations:** Department of Research Oncology, King's College London, Guy's Hospital Campus, Great Maze Pond, London SE1 9RT, UK

## Abstract

The role of magnetic nanoparticles (MNPs) in medical applications is rapidly developing. Advances in nanotechnology are bringing us closer to the development of dual and multifunctional nanoparticles that are challenging the traditional distinction between diagnostic and treatment agents. The current use of MNPs in breast cancer falls into four main groups: (1) imaging of primary and metastatic disease, (2) sentinel lymph node biopsy (SLNB), (3) drug delivery systems, and (4) magnetic hyperthermia. The current evidence for the use of MNPs in these fields is mounting, and potential cutting-edge clinical applications, particularly with relevance to the fields of breast oncological surgery, are emerging.

## 1. Introduction

Magnetic nanoparticles (MNPs) possess unique properties, which make them highly attractive to medical applications. These properties include their high surface to volume ratio, their quantum properties, and their ability to carry other compounds due to their small size [1]. MNPs are widely abundant in biological systems, ranging from geomagnetic navigational aids in migratory species to ferritin which is the most common iron storage protein and which contains up to 3000 ferric ions in a paramagnetic oxyhydroxide core [2]. The microscopic origin of magnetic properties in matter lies in the orbital, and spin motions of electrons whose spin and angular momentum are associated with magnetic moment [3]. The interaction between the magnetic moments of atoms from the same material causes magnetic order below a certain critical temperature. Bulk materials are classified on the basis of these interactions and their influence on material's behaviour in response to magnetic fields at different temperatures. If the volume of a material is reduced as in the case of MNPs, to just one domain, the magnetic properties are no longer similar to bulk materials [4].

Due to their small volume, MNPs usually present with superparamagnetic behaviour. This means that thermal energy may be enough to change spontaneously the magnetization within each MNP by allowing the magnetic moment of each MNP to rotate randomly just because of temperature influence. For this reason, in the absence of an electromagnetic field the net magnetic moment of a system containing MNPs will be zero at high enough temperatures. However, in the presence of a magnetic field, there will be a net statistical alignment of magnetic moments except that now the magnetic moment is that of the MNPs-containing various atoms which can be up to 10^4^ times larger than for a paramagnetic material. The lack of remanent magnetization after removal of an applied external field enables the MNPs to maintain their colloidal stability and avoid agglomeration. This property is important for biomedical applications [4].

MNPs with a hydrodynamic diameter of less than 5 nm quickly extravasate across the endothelium and have short blood circulation times. MNPs smaller than 6 nm in size undergo glomerular filtration and renal clearance. MNPs over 8 nm, and those with specific surface properties such as charge and hydrophobicity, are phagocytosed by liver Kupffer cells and undergo clearance via the biliary system [5]. Some small MNPs escape opsonisation by the reticulo-endothelial system and can be large enough to be retained within the systemic circulation. These characteristics, along with the enhanced permeability and retention effect (EPR) demonstrated by Maeda, lead to longer circulation times of these particles in the body [6]. EPR arises as a result of the production of vascular endothelial growth factor (VEGF) by the tumour. VEGF promotes disorganized angiogenesis leading to the production of “leaky” blood vessels with permeable walls [7]. Many tumours lack effective lymphatic drainage. Both these factors lead to the retention and accumulation of MNPs, 10–100 nm in size, at the tumour site, making MNPs ideal for localized diagnostic and imaging applications.

The modern treatment of solid cancers has now become more tailored to the individual patient and to specific tumour types. Surgery has become more tailored and in general, more conservative. MNPs provide an opportunity for the development of the next generation of focused diagnostic and therapeutic oncological applications. The development of focused therapy can reduce the side effect profile associated with current nonspecific cancer therapies. This could be achieved by reducing the systemic effects of chemotherapeutic drugs and radiotherapy, reducing the invasiveness of procedures, the morbidity associated with incomplete excision of lesions, and the inability to identify the presence of malignant lesions on imaging.

Currently, there are 4 main areas in which MNPs are being developed for breast cancer therapies: firstly, molecular imaging with targeted contrast agents for magnetic resonance imaging (MRI); novel techniques for sentinel lymph node biopsy (SLNB); magnetic hyperthermia and magnetic drug delivery systems. The current evidence for the use of MNPs is mounting, and potential cutting-edge clinical applications, particularly with relevance to the field of breast oncological surgery, are emerging.

## 2. The Role of MNPS in the Imaging of Malignancies

MNPs are established in aiding radiological imaging of malignant lesions, particularly with MRI. MNPs can be used for targeting the primary malignant lesion, assessing metastatic spread and particularly metastatic lymph node spread. 

### 2.1. MNPs in the Imaging of Primary Malignancies

Nanoparticles have been successfully used to selectively tag a wide range of medically important targets, including bacteria, biomarkers, and individual molecules such as proteins and DNA [8]. The application of MNPs to the localization of breast cancers on imaging has been focused upon their conjugation with receptor, binding ligands. Particular importance has been placed upon conjugation with HER2 monoclonal antibody (HER2 Ab) which allows receptor specific binding to HER2 (present in about 17% of breast cancers). The use of HER2 as a target for MNP-HER2 Ab has been applied both *in vitro* and *in vivo*. Oghabian et al. [9] used superparamagnetic iron oxide (SPIO) nanoparticles (30 nm) characterised as superparamagnetic in properties and conjugated with HER2 Ab as a MRI contrast agent in an induced tumour xenograft model of BT474 breast cancer cells in female nude mice. The HER2 Ab conjugated iron oxide nanoparticles were demonstrated to be efficient for *in vivo* diagnosis on T2-weighted images MRI. Chen et al. [10] also formed a conjugate of iron oxide and Herceptin-dextran nanoparticles. It was demonstrated that Herceptin-nanoparticles specifically targeted the HER2/neu receptor expressing tumours in a mouse xenograft model as seen on T2 weighted MRI. Funovics et al. [11] conjugated monocrystalline iron oxide nanoparticles with antibodies against HER2/neu receptor. It was demonstrated that all receptor positive breast cancer cell lines showed receptor-specific immune fluorescence and strong changes in T2 signal intensity. Artemov et al. [12] used SPIO conjugated with Herceptin to visualize breast cancer cell lines (MCF-7, MDA-MB-231, and AU-565). Strong negative T2 contrast was detected in all cell lines probed with specific Herceptin mAb demonstrating their potential qualities as an MRI contrast agent. Ma et al. [13] used the formation of magnetic nanoprobes to act as T2 contrast agents for MRI to image breast cancer. The multilayered, core/shell nanoprobes (MQQ-probe) contained Fe_3_O_4_ MNPs, visible-fluorescent quantum dots (600 nm emission), and near infrared-fluorescent QDs (780 nm emission) in multiple silica layers. To demonstrate the function of the MQQ probe *in vitro*, cellular imaging was conducted for human breast cancer cells (GFP-expressing KPL-4 cancer cell line). Anti-HER2 antibody was conjugated to the MQQ-probe (HER2-MQQ-probe). The targeting ability of the HER2-MQQ-probe to the tumour cells was examined using fluorescence microscopy, and HER2-MQQ-probes were found on the surface of the tumour cells effectively. The viability of cells decreased with an increasing concentration of the probe and incubation time. The HER2-MQQ-probe was assessed *in vivo* in a breast cancer tumour model (transplantation of GFP-expressing KPL-4 cells in nude mice). The multimodality nature of the probe was assessed using NIR fluorescence and MRI. NIR fluorescence distinguished breast tumour from surrounding tissues 48 hours after HER2-MQQ-probe was injected. T2 weighted MRI demonstrated detailed tumour morphology at 48 hours after injection. MQQ probes can thus be used as both NIR fluorescent probes and as MRI contrast agents.

Other conjugates have also been developed for imaging purposes. Lim et al. [14] created hyaluronan-modified magnetic nanoclusters (HA-MNCs) for the detection of CD44 over expressing breast cancer using MRI. The magnetic nanoclusters are composed of pyrenyl HA (Py-HA) conjugates and are capable of simultaneously encapsulating magnetic nanocrystals (iron-based) and targeting CD44 using Py-HA. The targeting efficiency of the HA-MNCs was investigated by MRI against MDA-MB-231 cells (high CD44 expression) and MCF-7 cells (low CD44 expression). T2 weighted MRI demonstrated that HA-MNCs possessed a remarkably strong MRI contrast enhancement effect on MDA-MB-231 cells expressing CD44. This was then investigated *in vivo* by injection of MDA-MB-231 cells into the mammary fat pads of nude mice as an orthotopic breast cancer mouse model. Postinjection tumour sites distinctly acquired immediate changes associated with high MRI signal intensity. HA-MNCs, therefore, demonstrated clear potential to act as MRI contrast agents. Campbell et al. [15] evaluated magnetite/silica core-shell nanoparticles (Mag@SiO_2_) and their applicability to use as a contrast agent for MRI in biological tissues. *In vivo* MRI in a breast tumour mouse model demonstrated T2 signal enhancement at the tumour site following administration of Mag@SiO_2_. Prashant et al. [16] used SPIO loaded in a copolymer of poly (lactic acid) (PLA) and D-alpha-tocopherol polyethylene glycol 1000 succinate (TPGS) as a contrast agent for MRI. These SPIO-loaded PLA-TPGS (SPIO-PNPs) underwent qualitative investigation of cellular uptake in MCF-7 breast cancer cells by TEM visualization. *In vivo* mice xenograft models demonstrated internalization of the SPIO-PNPs by the tumour cells, 5 hours after SPIO-PNP administration. The SPIO-PNP also accumulated in the liver. However, the signal intensity within the liver was lost within 24 hours demonstrating an advantage over current SPIO such as Resovist (Schering, Germany). Jain et al. [17] used Pluronic-stabilized iron oxide nanoparticles as MRI contrast agents. The copolymer MNP was then introduced into a xenograft mouse model. This showed that selected F127-modified MNPs displayed MRI contrast enhancement in both the vascular tumour periphery and in the whole tumour at one hour. Yang et al. [18] in their study created receptor targeted nanoparticles for *in vivo* imaging of breast cancer. A novel iron oxide nanoparticle was created using a recombinant peptide containing the amino-terminal fragment of urokinase-type plasminogen activator (uPA) conjugated to magnetic iron oxide nanoparticles amino fragment conjugated-iron oxide (ATF-IO). This nanoparticle targets uPA receptor which is overexpressed in breast cancer tissues. The ATF-IO nanoparticles bound to the uPA receptor proteins on the breast cancer cell lines and were internalized. ATF-IO selectively accumulated in tumours on *in vivo* studies as demonstrated by a reduction in T2 values and signal decrease in T2 weighted images in various areas of the tumour mass.

The combination of radionuclide-based imaging modalities such as single photon emission computed tomography (SPECT) and positron emission tomography (PET) with magnetic resonance imaging (MRI) is being developed as the next generation of clinical scanners. This is because radionuclide-based techniques (SPECT/PET) are extremely sensitive to changes in tumour function and allow us to study processes at the molecular and cellular level *in vivo*. However, their spatial resolution is very poor (>10 mm). Conversely, nonradionuclide-based techniques such as computed tomography (CT) and MRI provide excellent spatial resolution (<1 mm), therefore growing interest exists in the development of SPECT and PET-MRI agents. de Rosales et al. [19] conjugated radiolabelled bisphosphanate (BP) directly to the surface of SPIO nanoparticles, in the form of ^99m^Tc-dipicolylamine- (DPA-) alendronate, a BP-SPECT agent, and Endorem (Guerbet, France), a liver MRI contrast agent based on SPIO. The bimodal imaging capabilities and excellent stability of these nanoparticles were confirmed using MRI and nano-SPECT-CT imaging, showing that ^99m^Tc and Endorem colocalize to the liver and spleen *in vivo*, as expected for particles of the composition and size of ^99m^Tc-DPA-ale-Endorem.

### 2.2. MNPs in the Imaging of Metastatic Disease

Metastatic disease accounts for most of cancer mortality. The 5 year survival rate of breast cancer patients sharply decreases from 98% in cases with localized primary lesions to 23% in cases of distant metastases [20]. The inability to diagnose small volume metastases early on in the disease process has limited effective treatment of metastatic breast cancer [20]. The imaging of metastatic disease poses some unique problems. Current nanoparticle delivery systems have been developed to make use of the “enhanced permeabiltiy and retention effect” (EPR) of the tumour microenvironment. This means that due to tumour's leaky vasculature, the intratumoural accumulation of nanoparticles is high relative to normal tissues. Whilst the EPR strategy is effective for large, well-vascularized primary tumours, it is not the case in smaller metastatic clusters which are present within a range of tissue types. Targeting a metastatic lesion within a large population of normal cells presents a unique challenge. Metastases present biobarriers due to their smaller size, higher dispersion to organs, and lower vascularization than primary tumours, making them less accessible to molecular or nanoparticle agents [21]. A range of conceptual techniques have been developed as potential targets. Peiris et al. [22] considered the use of nanoparticles designed for targeting alpha _v_ beta _3_ integrins. Beta _3_ integrins have been shown to be responsible for migration, invasion, and metastasis. It has been shown that the metastatic site transitions from selectin-dependent tumour cell rolling on the endothelium to firm attachment that is mediated primarily by alpha _v_ beta _3_ integrins, therefore making alpha _v_ beta _3_ integrins a desirable target. They designed a nanoparticle composed of 4 iron oxide nanospheres chemically linked with cyclic tripeptide arginine-glycine-aspartic acid (cRGD) peptide as the ligand for targeting the nanoparticles to metastases (RGD-NC). RGD-NC was applied to a late stage 4-tumour model in mice. MRI demonstrated that the RGD-NC nanoparticles targeted metastatic lesions by achieving a significantly higher signal drop aiding identification within the liver parenchyma. Histological evaluation of the liver confirmed preferential accumulation of the agent within the metastatic lesions.

Kievit et al. [23] used SPIO coated in a copolymer of chitosan and PEG (NP) (which aids anchoring for drugs, imaging agents and provides steric increased colloidal stability and decreased immune recognition) with neu antibody (HER2 Ab) to target metastatic breast cancer. MRI studies on transgenic mice demonstrated that targeted NP-neu significantly shortened T2 relaxation time making it suitable as a MRI contrast agent for disease detection or monitoring of drug delivery. Histology from lung and liver micrometastases from NP-neu treated mice showed significant positive iron staining. Targeting of micrometastases was much more pronounced than primary tumour targeting when comparing targeted (NP-neu) which is likely due to the lack of the EPR effect in early stage micrometastases. Large tumours contain leaky vasculature allowing large influx of blood including nutrients and oxygen and have insufficient lymph vessels to remove waste. This leads to the EPR effect which can be used for passive targeting of tumours with nanoparticles. However, micrometastases, which can currently only be identified histologically, are still small enough where diffusion provides adequate oxygen, so tumour angiogenesis is not yet induced by anoxic conditions. Unlike the large tumours, lymphatic drainage from the lungs and liver and hepatic ducts of the liver are adequate to remove waste. Therefore, these metastases do not have the enhanced retention associated with large tumours and thus cannot be passively targeted. This explains why a more significant targeting effect was seen in these metastases by targeted delivery [23].

Nacev et al. [21] looked at techniques to increase MNP concentrations within metastatic lesions as opposed to surrounding healthy tissues. They reviewed postmortem specimens of 18 women who died from metastatic breast cancer and analyzed histologically their lungs and livers. They found that the tumour vessels were generally larger in diameter but fewer in number than in the adjacent normal liver, with a more random distribution and a greater vessel-to-vessel spatial separation. They postulated that systemically delivered nanoparticles would preferentially accumulate within benign tissue. Nacev et al. [21] created a model simulation based upon the post-mortem specimens and previous clinical data to assess dynamic magnetic shift (DMS). This involves an external alternating magnetic field being applied in regions of metastatic disease to focus MNPs for diagnostic and therapeutic purposes. Alternating the direction of magnetic forces more effectively normalized particle concentration between normal and tumour tissue as the time averaged concentration of particles in the tumour was 18.0%, which is close to the 19.7% concentration in normal tissue, a 1.99-fold improvement compared with no magnetic actuation. Therefore, DMS may play a role in allowing therapeutic MNPs to reach areas of poorly vascularized metastatic tumours more effectively than by diffusion alone.

### 2.3. MNPs in Assessment of Metastatic Lymph Node Spread

MNPs have been the focus of much attention for imaging lymph nodes. MNPs have been traditionally used as contrast agents for MRI, as they alter magnetic field gradients therefore altering proton relaxation in the imaged tissue. SPIO used as contrast agents are usually injected intravenously, allowing them access to the nodes by moving into the medullary sinuses of the node via direct transcapillary passage, as well as directly accessing nodes via lymphatic flow as with interstitial injection. SPIO transported to the SLN act as “negative” imaging contrast agents when using T2 and T2* weighted pulse sequences and have been shown to identify metastases independently of lymph node size [17]. As SPIO is filtered and taken up by macrophages, healthy lymph node tissue produces a dark signal using a T2/T2* sequence. If the lymph node is metastatic, the node will show up as bright (or partially bright), as the unhealthy node lacks macrophage activity. This technique allows for a noninvasive method of imaging the complete lymphatic drainage pathway from the tumour, and then determining the involvement and location of any clinically positive lymph nodes [24, 25]. Johnson et al. [26] took this further and histologically assessed the excised sentinel lymph nodes (SLNs) of patients who had SPIO administered and subsequent SLNB. They found that of the 13 involved nodes iron was not deposited at the site of the metastases except in one case. This drew them to conclude that heterogenous enhancement of SLNs on MRI may indicate a metastatic focus. SPIO when injected intravenously have been shown to provide the best sensitivity and specificity for the detection of the involved node on MRI [27].

The use of MRI with SPIO contrast agents for the staging of the axilla in breast cancer could potentially reduce the invasiveness of diagnostic and therapeutic procedures. Madru et al. [28] conducted a study on rats to assess the identification of the SLN on MRI using SPIO as a contrast agent. Their small study of 5 rats demonstrated SPIO uptake in all SLNs on histology and their identification on MRI. Numerous studies have looked at comparing preoperative MRI using SPIO as contrast agents to histological findings on SLNB. Motomura et al. [25] conducted their study upon 102 patients with breast cancer comparing preoperative MRI findings using SPIO as a contrast agent with histological findings on SLNB. They found that the sensitivity, specificity, and accuracy of MRI for the diagnosis of SLN metastases were 84.0%, 90.9%, and 89.2%, respectively. Harada et al. [24] found the results of conventional MRI to be a 59.1% sensitivity, 86.7% specificity, and 80.4% overall accuracy, whereas postcontrast SPIO figures were 100.0% sensitivity, 80.0% specificity, and 93.9% overall accuracy. Stadnik et al. [29] found a sensitivity of 100%, specificity of 80%, positive predictive value of 80%, and negative predictive value of 100% were achieved for SPIO enhanced MRI. Michel et al. [30] found that the results of axillary staging using SPIO enhanced MRI imaging were 9 true-positives, 7 true-negatives, zero false-positives, and false-negatives in 2 of 18 patients (sensitivity, 82%; specificity, 100%; positive predictive value, 82%). A systematic review considering the use of MRI assessment of axillary lymph node status in early breast cancer was performed by Cooper et al. [27]. This considered 9 studies, the largest of which contained 67 patients. They recorded a mean sensitivity of 90% (95% CI: 78–96%; range 65–100%) and mean specificity of 95% (95% CI: 75–96%; range 54–100). The highest mean sensitivity and specificity being recorded for ultrasmall paramagnetic iron oxide (USPIO) enhanced MRI, with values of 98% (95% CI: 61–100%) and 96% (95% CI: 72–100%), respectively. Kimura et al. [31] found that MRI lymphangiography with USPIO performed on 10 patients with breast cancer was able to accurately predict all metastatic and normal nodes preoperatively. Therefore, such studies do offer promising results for the future of MRI in this field. It is therefore essential that we develop further imaging studies to identify standardized criteria for MRI parameters that define metastatic axillary lymph nodes in breast cancer. The current SentiMAG multicentre trial, [32] which is a prospective phase II nonrandomized clinical trial to compare SLNB using magnetic nanoparticles versus the standard technique and is coordinated from Guy's Hospital, has an MRI subprotocol. This will evaluate preoperative axillary MRI (with SPIO magnetic tracer) for SLN imaging and characterization and *ex vivo* MRI with a 9.4 T high resolution scanner to identify metastases. The aim of the sub protocol is to evaluate MRI for both preoperative and intraoperative detections of lymph node metastases. The results from such studies will allow us to move forward to a truly minimally invasive approach to the future axillary management of breast cancer. MRI lymphangiography has the promise to eliminate the need for diagnostic surgical axillary procedures such as SLNB. The current issues existing, however, are the limited availability of MRI and its operational costs. Cooper et al. [27] performed a study looking at the cost-effectiveness of MRI and PET for the evaluation of axillary lymph nodes in early breast cancer. They created an individual patient discrete-event simulation model to estimate the lifetime costs and benefits of replacing SLNB with MRI or PET or adding MRI or PET before SLNB. Their findings suggest that the most cost-effective strategy predicted was the strategy of replacing SLNB with MRI. This strategy dominates the baseline SLNB strategy with both lower expected total costs (*£*19,325 versus *£*20,189) and higher expected total QALYs (8.174 versus 8.119). Under this strategy, true positive patients (39.3% of all patients) will be correctly diagnosed by MRI and undergo ALND rather than undergo two sequential surgical procedures (SLNB followed by ALND). True negative patients (52.5% of all patients) will be correctly diagnosed without the need for SLNB. The difficulty of applying this on clinical grounds arose in the study due to the false positive rate for MRI being 6.3% versus 0.2% for the current baseline strategy of SLNB. The technique of MRI lymphangiography has not yet been developed sufficiently to achieve the specificity and positive predictive value of SLNB. However, with future improvements it may have the potential to eradicate the need for SLNB altogether.

## 3. MNPs and Sentinel Lymph Node Biopsy

Cancer staging is not only a key predictor of survival but also critical for establishing the sequence of additional treatment required. Solid organ tumours such as breast cancer spread predominantly via the lymphatic system. When the cancer spreads, its cells migrate from the tumour and are carried away by the interstitial fluid in the lymphatic system [33]. This therefore makes knowledge of the regional lymph node status essential for providing information regarding staging local control and prognostic outcomes.

Until recently the regional lymph node status had been determined in breast cancer by axillary lymph node dissection (ALND). This changed in breast cancer with the pioneering work of Krag et al. [34] in 1993 and Giuliano et al. [35] in 1994, which led to the development of the technique of SLNB. SLNs are defined as the first lymph nodes that receive lymphatic drainage from the primary tumour. These nodes are the most likely to harbour metastatic cancer via lymphatic spread. SLNB is now regarded as the standard of care in patients without clinical evidence of nodal metastasis in early-stage breast cancer.

The current SLNB protocol involves injecting a technetium-sulphur colloid ([^99m^TC]-TSC) alongside isosulfan blue (N-[4-[4-(diethylamino) phenyl] (2,5-disulphophenyl) methylen]-2,5-cyclohexadien-1-ylidene]-N-ethylethanamine hydroxide), the so-called combined technique, interstitially into the breast around the tumour [36]. In the United Kingdom and some other countries, Patent Blue (Guerbet, France) is used instead of isosulfan blue. After allowing for the materials to localize in the lymphatic system, the clinician uses a handheld scintillation counter (gamma probe) to locate the nodes-receiving primary drainage from the tumour via lymphatic vessels. The blue dye assists in the localization after-incision, and lymph nodes that are radioactive, blue, or both are judged to be SLNs. The SLNs are then removed and sent for microscopic histological examination. The detection rate of the technique has been demonstrated to be 96% with a false negative rate of 7.3% on meta-analysis of over 8000 patients [37]. While effective, this procedure has a number of limitations. ^99m^TC has a 6-hour half-life which limits its availability to proximal centres that handle its parent isotope ^99^Mo. ^99^Mo decays so quickly that it must be supplied to hospital nuclear medicine departments every 2 weeks and is made in just a few reactors worldwide. ^99^Mo is the byproduct of nuclear fission and is therefore subject to interruption of supply pending refurbishment or compromise of nuclear facilities as was demonstrated in the Fukushima Daiichi nuclear disaster in Japan on March 11, 2011 [38].

For hospitals without ready access to radioisotopes, their use presents logistical issues requiring time and resources to be devoted to a nuclear medicine department for a routine procedure. Mandatory handling and waste disposal regulations, training, and licensing of operating theatre staff all add to overheads. The 6-hour half-life of the isotope limits theatre scheduling since the injection is performed by the nuclear medicine department and not by surgeons themselves. In addition patients may express reluctance to radiation exposure especially in the pregnant state [38]. These factors have been a significant barrier to the uptake of SLNB worldwide for hospitals around the world without access to radioisotopes. While the incidence of cancer is rising the SLNB procedure has plateaued with only around 60% of an estimated 500,000 patients in the western world having access to the procedure [39]. This figure drops to 5% in China and is minimal in the rest of the world [40].

MNPs exhibit potential to replace the “combined technique” in SLNB due to some of their shared features. Firstly, they can be externally detected in tissue, preincision, secondly, they are of similar dimensions to the radiotracer colloid, and thirdly, their brown-black appearance acts as a visual stain. MNPs have been under clinical investigation and development as diagnostic and imaging agents for the last 40 years. The recent focus has been upon iron oxides such as maghemite (gamma Fe_2_O_3_) and magnetite (Fe_3_O_4_) coated in a biocompatible molecule such as dextran to form SPIO. Below 30 nm in diameter they exhibit superparamagnetic behaviour. This makes them ideal for SLNB, as they do not agglomerate while being transported via lymph in the absence of an external field. In the presence of a static or dynamic external field, however, their collective moment can be sensed external to the body [41].

The benefits of SPIO include that they have a half-life of several years allowing them to be shipped to remote locations worldwide. They do not require special handling procedures, which therefore allow the surgeon to inject them, removing issues of scheduling with a nuclear medicine department as well as problems with safe waste disposal. SPIO are not believed to be toxic or dangerous in clinical use. SPIO have a half-life of between 1 and 36 hours depending upon their coating and whether they are injected intravenously or interstitially before they are taken up by macrophages in the mononuclear phagocyte system of the liver, spleen, lymphatic, and bone marrow and broken down to be distributed across iron stores in the body [42]. SPIO have been used as MRI contrast agents with doses of iron oxide around 25–100 mg. Such a dose is roughly equivalent to 2–5 days of normal dietary intake of iron, which results in transient changes in serum iron, ferritin, and iron-binding capacity but does not risk iron overload. Studies on the toxic effects from SPIO at increased dosages confirmed no acute or sub-acute toxic effects in rats or beagles that received 150 times the standard dosage for MRI of the liver. Although the coating of the SPIO could be a potential source of allergic reaction, currently most SPIO are coated in dextran or carboxydextran with no allergic reactions being reported for these materials [33, 43].

The current NIHR registered SentiMAG multicentre trial [32] co-ordinated from Guy's Hospital, London is a prime example of how MNPs can be applied to the performance of SLNB in breast cancer. This trial is a phase II, non-randomized, multicentre, equivalence study comparing the standard combined technique SLNB in breast cancer using blue dye and ^99m^Tc with SLNB performed using SPIO (Sienna+; Endomagnetics, UK) and a handheld magnetometer (SentiMag; Endomagnetics, UK). An initial proof of concept study was performed at University College London by Joshi et al. [44] in which a total of 19 SLNs were resected from 9 patients with breast cancer. Intraoperative localization using the combined technique was successful in detecting all 19 SLNs and using the SentiMag prototype all 19 SLNs were also identified. This was followed by an extended phase I/II trial whose results suggested that the SLN detection rate using the new technique was 86% (37/43 patients) and 93% (14/15 patients) when the SPIO was administered more than 1 hour prior to surgery, suggesting that a higher volume or concentration of SPIO was required [44].

All patients requiring SLNB are eligible for the SentiMag trial and will receive standard radioisotope, SPIO and patent blue dye. All SLNs detected intraoperatively using the gamma probe and handheld magnetometer will be excised. All lymph nodes will be assessed histologically and the node status related back to the SLNB detection rate with each technique. The aim is to compare the SLN detection rate and performance of the new technique with that of the standard technique. Primary endpoints are the proportion of SLNs detected (detection rate) with both the standard and new technique. Secondary endpoints will include morbidity from SLNB such as lymphoedema, numbness, seroma, cutaneous staining, shoulder stiffness, chronic pain and loco-regional recurrence. Currently over 350 patients have been recruited and the initial data analysis on this group of patients is currently underway. Pending results, progression to a randomized control trial is scheduled.

Magnetic SLNB is not just applicable to breast cancer. Minamiya et al. [45] conducted a study on patients with nonsmall cell lung cancer. Each patient received 5 mL of ferumoxides injected around the tumour and the magnetic readings recorded using a handheld magnetometer. In their small study of 20 patients, they found the SLN detection rate to be 80% (16/20). Nakagawa et al. [46] also performed the procedure on 38 patients with nonsmall cell lung cancer using SPIO of nonstoichiometric magnetite. They found a SLN detection rate of 81.6% (31/38). *Ex vivo* studies have also been conducted on colorectal cancer specimens by ten Haken et al. [47]. They took postresected colorectal specimens, injected SPIO around the tumour site, and then used the handheld magnetometer to identify SLNs which were excised. They were able to identify the SLNs using the magnetometer, and histopathologically they were able to identify the SPIO in the resected SLNs. 

 Another trial coordinated from Guy's Hospital is comparing the current combined SLNB technique with the magnetic technique in patients with malignant melanoma. The Technology Strategy Board funded MELAMAG study [48] will be a multicentre, nonrandomized, and equivalence study comparing detection rate between the two techniques together with associated morbidity.

## 4. MNPs as Drug Delivery Systems

MNPs provide an opportunity to develop drug delivery systems which are specifically designed for their target. Current chemotherapeutic regimens suffer from nonspecific toxicities which limit their therapeutic potential. Numerous studies have looked at mechanisms in order to optimize MNPs for selective, targeted drug delivery. However, MNP use still has issues to be addressed which include drug-loading capacity, desired release profile, aqueous dispersion stability, biocompatibility with cells and tissue, and retention of magnetic properties after modification with polymers or chemical reaction.

Numerous studies have been conducted on the release of chemotherapeutic drugs *in vitro*. Such studies have focused upon creating MNP conjugates which allow specific targeting with antiproliferative cancer effects. These studies have demonstrated success on an *in vitro* scale. Jain et al. [49] developed a novel water-dispersible oleic acid- (OA-) Pluronic-coated iron oxide magnetic nanoparticle formulation that could easily be loaded with high doses of water-insolute chemotherapeutic drugs. They demonstrated internalization of DOX-loaded nanoparticles in MCF-7 cells with sustained intracellular retention and dose-dependent antiproliferative activity in cancer cells. Kohler et al. [50] developed a drug-nanoparticle conjugate by grafting methotrexate (MTX) to the iron oxide nanoparticle. They modified the surface of the MNP conjugate via a peptide bond preventing MTX being released from the surface of the nanoparticles under intravenous conditions. Cleavage of the amide bond occurs under conditions present in the lysosomal compartment. Such an environment is characteristically a low pH and in the presence of lysozymes which is characteristic of the environment within the target cells. Due to the overexpression of folate receptors on target cells as opposed to healthy cells increased uptake of MTX conjugated nanoparticles in tumour cells was demonstrated. Munnier et al. [51] developed aqueous suspensions of SPIO by coprecipitation of ferric and ferrous chlorides in alkaline medium followed by surface oxidation by ferric nitrate and surface treatment with citrate ions. The ferrofluids were loaded with DOX using a pre-formed DOX-Fe^2+^ complex. By using this technique loading values as high as 14% were achieved. The *in vitro* cytotoxicity of the DOX-loaded SPIO on the MCF-7 breast cancer cell line was similar to that of DOX in solution or even higher, at low-drug concentrations. Benyettou et al. [52] created a delivery system for zoledronate by conjugating it with SPIO. The anchoring to nanoparticle's surface allowed an increase in their hydrophobicity and also to change the therapeutic target, increasing the Zoledronate intestinal absorption instead of their accumulation in bone. In addition to this, *in vitro* studies on MD-MB 231 showed that the conjugate had antiproliferative activity. Yallapu et al. [53] developed MNPs with an iron oxide core coated in oleic acid (OA) and then with OA-PEG (polyethylene glycol) to form a water dispersible MNP formulation. Doxorubicin hydrochloride was converted to a water-insoluble base (DOX), so that it could partition into the OA layer. *In vitro *studies on MCF-7 breast cancer cells demonstrated dose-dependent antiproliferative effects. Gautier et al. [54] created a PEG-SPIO which was loaded with DOX which demonstrated cytotoxicity on MCF-7 breast cancer cells *in vitro*. Tong et al. [55] also used PEG-iron oxide conjugate for chemotherapeutic delivery. However, in this case conjugation was with gemcitabine to create a new delivery system of magnetic gemcitabine long-circulating liposomes (MGLL). *In vitro* MTT assay showed that MGLL had significant cytotoxicity to MCF-7 cells compared with the conventional modalities as well as in *in vivo* studies.

Other techniques for drug delivery have included magnetic hyperthermia. Hayashi et al. [56] developed MNPs based upon iron oxide with folate conjugate as a targeting ligand for breast cancer cells and cyclodextrin (CD) as a drug container (FA-CD-SPIO). FA-CD-SPIO were loaded with chemotherapeutic drugs, and a high frequency magnetic field (HFMF) applied. This resulted in a hyperthermic effect and the release of the therapeutic drug (TMX) from the CD component.

MNPs have also been used for transfection of cells. These techniques have relied upon coating SPIO with cationic ploymers, such as polyethylenimine (PEI). The high positive charge of PEI promotes DNA transfer into cells. Biswas et al. [57] synthesized a lipid coated magnetic nanoparticle formulation (dMLP). This was used to successfully transfect MCF-7 breast cancer cells. Kumar et al. [58] applied gene transfection to the delivery of siRNA into breast cancer cells. They synthesized a tumour targeted nanodrug (MN-EPPT-siBIRC5) that was designed to specifically shuttle siRNA into human breast tumours. MN-EPPT-siBIRC5 consists of SPIO, peptides (EPPT), and a synthetic siRNA that targets the tumour specific antiapoptotic gene BIRC5. Nanodrug uptake by human breast adenocarcinoma cells resulted in a significant downregulation of BIRC5 and increase in apoptotic nuclei present *in vitro* and *in vivo*. Following intravenous delivery into subcutaneous mouse models of breast cancer, the nanodrug demonstrated a preferential tumour uptake, which could be visualized by MRI and near-infrared optical imaging.

Alternative approaches to the treatment of cancer aside from delivery of chemotherapeutic agents to induce apoptosis or DNA/siRNA to regulate oncogene expression have been used. Veiseh et al. [59] looked at an approach of treatment through inhibition of cell invasion. They synthesized a conjugated MNP which comprised of an iron oxide nanoparticle core coated with an amine-functionalized PEG silane attached to cholorotoxin chemotherapeutic agent (CTX), forming CTX enabled nanoparticles (NPCs). The study demonstrated that NPCs had a higher affinity for tumour cells and therapeutic effect compared to unbound CTX and that gelatinase activity of MMP-2 in the presence of NPC demonstrated comparable inhibition.

One of the main alternative nanoparticles to MNPs that have been explored for drug delivery systems is gold. Gold has been used to deliver chemotherapeutic agents due to its high drug loading as demonstrated by Gibson et al. [60] who coupled over 70 molecules of paclitaxel to a gold nanoparticle with a 2 nm core diameter. Gold nanoparticle drug delivery systems function in 2 ways. Firstly, via glutathione- (GSH-) mediated release for intracellular activation. Studies have demonstrated cellular delivery and GSH-mediated release of a hydrophobic dye using functionalized gold nanoparticles [61]. Controlled release can be achieved by varying intracellular GSH levels by using a GSH-monoester which is hydrolyzed to GSH as demonstrated in embryonic fibroblast cells [62]. Secondly, gold nanoparticles can be employed by delivery of therapeutic agents such as singlet oxygen and nitric oxide. Polizzi et al. [63] showed that nitric-oxide can be stored by covalent linking with polyamine-stabilized gold nanoparticles and allowed for effective release of nitric oxide from the water soluble nanocontainers. Wieder et al. [64] attached phthalocyanines to the surface of gold nanoparticles which act as photosensitisers to generate singlet oxygen which is cytotoxic. These techniques allow for the targeting of drugs to specific organs or tissues using gold nanoparticles as a delivery system. However, the application of gold nanoparticles can also be extended to analytical and biological sciences including chemical sensing and imaging applications. Colorimetric sensors composed of gold nanoparticles can be used for heavy metal cation determination such as lead and mercury screening [65]. Gold particles can be used for the detection of DNA [66] and when conjugated with antibodies have been used with light and electron microscopy for visualizing proteins in biological samples [67]. They have been used as calorimetric indicators to evaluate enzymatic activities and screen enzyme inhibitors. Their possession of excellent biocompatibility and unique structural, electronic, magnetic, optical, and catalytic properties also make them attractive material for biosensor, chemisensor, and electrocatalysts [62].

## 5. The Role of MNPs in Magnetic Hyperthermia

Magnetic hyperthermia is a promising field in which MNPs are exposed to an alternating magnetic field (AMF) and generate heat due to magnetic hysteresis loss. If cancer tissue is in contact with MNPs, this can be therapeutic [68]. Numerous studies have looked at *in vitro* and *in vivo* applications of this technique. Rodríguez-Luccioni et al. [68] applied an AMF to magnetitie nanoparticles in MCF-7 breast cancer cell cultures and demonstrated significant reduced viability of cells compared to hot water-induced hyperthermia. Kikumori et al. [69] also used magnetite nanoparticles but in an *in vivo* mouse model. Here BT474 (High HER2 expression) and SKOV3 (low HER2 expression) cancer nodules were introduced into the* in vivo* model. An AMF was applied with the tumour temperature increased to 45°C and tumour regression observed in the hyperthermic group and was sustained for 10 weeks after-treatment. Hilger et al. [70] used an immunodeficient SCID mouse model with intratumoral injection of iron oxide particles and the application of an AMF. Histological examination demonstrated the early stages of coagulation necrosis in treated tumour cells.

Conjugation of antibodies to MNPs has been used for targeted magnetic hyperthermia. Ito et al. [71] conjugated magnetite nanoparticles with anti-HER2 immunoliposomes and applied these to a culture of SKBr3 breast cancer cells *in vitro*. A subsequent AMF was applied in order to induce magnetic hyperthermia. The combination of anti-HER2 antibody therapy with hyperthermia resulted in strong cytotoxic and antiproliferative effects. The conjugation of MNPs to specifically targeted antibodies can also be used to target metastatic cancer. DeNardo et al. [72] produced a 111In-ChL6 bioprobes (ChL6 is chimeric L6) in a human breast cancer xenograft model. The application of an AMF after bioprobe administration confirmed tumour growth delay compared to controls, suggesting that mAb-guided bioprobes (iron oxide nanoparticles) effectively targeted human breast cancer xenografts in mice.

## 6. The Future Surgical Application of MNPs to Breast Lesion Localization

The surgical applications of MNPs are not confined to the identification of SLNs. Indeed, potential exists for their use in the pre- and intraoperative localization of nonpalpable lesions such as screen-detected breast cancer. These are occult, nonpalpable breast lesions which are identified by mammographic screening programmes. Worldwide 4.9 million breast biopsies annually are performed, and 3.2 million of these are performed for screen detected, nonpalpable breast lesions, of which a third are found to be malignant [38]. The current treatment regimen for these patients involves a 3-stage procedure of breast biopsy, followed by wire-guided localization and subsequently surgical removal. However, the use of a handheld magnetometer and specialized MNP delivery system could well avoid the need for a second localization procedure in this one million patients. At the performance of the initial breast biopsy a ferromagnetic clip loaded with MNPs could potentially be introduced into the biopsy cavity. Confirmation of the positioning of the magnetic clip could be done on postprocedure mammography. In those patients with a confirmed malignancy, patients could be delivered to the operating theatre without a second localization procedure for wire insertion. Intraoperatively the surgeon could be guided by the handheld magnetometer and MNP loaded magnetic clip in order to localize the breast cancer and excise it completely. Localization of lesions could be further enhanced by conjugation of MNPs with Herceptin directed specifically against the genetic profile of breast cancers that are Her2 positive such as in numerous previous studies using Herceptin conjugated MNPs to localize breast lesions *in vitro* and *in vivo* [9–14]. Therapeutic modalities could also be applied on administration of MNPs. MNPs could be conjugated with chemotherapeutic agents in order to commence localized primary systemic therapy (PST) prior to any surgical intervention as standard. Alternatively, the application of an alternating magnetic field to MNPs could be used in order to induce magnetic hyperthermia and partially or completely destroy small occult lesions limiting the extent of or completely avoiding the need for surgical intervention. Preclinical trials in order to overcome the difficulties of developing a consistent delivery system with nonmigratory marker need to be conducted. Such technology could easily be applied to the treatment of other cancers such as colorectal malignancies. At screening colonoscopy suspicious lesions such as small polyps could be marked with a magnetic clip loaded with MNPs. On confirmation of malignancy intraoperative guidance of the surgeon could be achieved using a handheld magnetometer at both open and laparoscopic surgeries.

## 7. Conclusion

MNPs have been used to localize nonpalpable lesions in a variety of ways for imaging, drug delivery, and magnetic hyperthermic purposes. These areas continue to advance rapidly. However, the surgical applications of MNPs are now becoming realized in terms of SLNB for breast cancer and malignant melanoma. Future preclinical work is now underway in order to further develop techniques of non-palpable breast lesion localization using ferromagnetic clip technology loaded with MNPs and handheld magnetometers in order to directly aid the surgeon intraoperatively to excise these lesions using this novel technique. Further applications of magnetic hyperthermia, localized PST, and Herceptin therapy may yet be realized for breast cancer and other solid tumours.
